# Plant-Based Products Originating from Serbia That Affect P-glycoprotein Activity

**DOI:** 10.3390/molecules29184308

**Published:** 2024-09-11

**Authors:** Jelena Dinić, Ana Podolski-Renić, Miroslav Novaković, Liang Li, Igor Opsenica, Milica Pešić

**Affiliations:** 1 Institute for Biological Research “Siniša Stanković”—National Institute of the Republic of Serbia, University of Belgrade, Despota Stefana 142, 11108 Belgrade, Serbia; jelena.dinic@ibiss.bg.ac.rs (J.D.); ana.podolski@ibiss.bg.ac.rs (A.P.-R.); 2 Institute of Chemistry, Technology and Metallurgy—National Institute of the Republic of Serbia, University of Belgrade, Njegoševa 12, 11000 Belgrade, Serbia; mironov@chem.bg.ac.rs; 3 Key Laboratory of Bioactive Substance and Function of Natural Medicines, State Key Laboratory of Respiratory Health and Multimorbidity, NHC Key Laboratory of Biotechnology for Microbial Drugs, Department of Oncology, Institute of Medicinal Biotechnology, Chinese Academy of Medical Sciences and Peking Union Medical College, No.1 Tiantan Xili, Beijing 100050, China; liliang@imb.pumc.edu.cn; 4 Faculty of Chemistry, University of Belgrade, Studentski trg 12–16, 11158 Belgrade, Serbia; igorop@chem.bg.ac.rs

**Keywords:** P-glycoprotein, multidrug resistance, cancer, natural products, natural product derivatives

## Abstract

Our review paper evaluates the impact of plant-based products, primarily derived from plants from Serbia, on P-glycoprotein (P-gp) activity and their potential in modulating drug resistance in cancer therapy. We focus on the role and regulation of P-gp in cellular physiology and its significance in addressing multidrug resistance in cancer therapy. Additionally, we discuss the modulation of P-gp activity by 55 natural product drugs, including derivatives for some of them, based on our team’s research findings since 2011. Specifically, we prospect into sesquiterpenoids from the genera *Artemisia*, *Curcuma*, *Ferula*, *Inula*, *Petasites*, and *Celastrus*; diterpenoids from the genera *Salvia* and *Euphorbia*; chalcones from the genera *Piper*, *Glycyrrhiza*, *Cullen*, *Artemisia*, and *Humulus*; riccardins from the genera *Lunularia*, *Monoclea*, *Dumortiera*, *Plagiochila*, and *Primula*; and diarylheptanoids from the genera *Alnus* and *Curcuma*. Through comprehensive analysis, we aim to highlight the potential of natural products mainly identified in plants from Serbia in influencing P-gp activity and overcoming drug resistance in cancer therapy, while also providing insights into future perspectives in this field.

## 1. Drug Resistance in Cancer Therapy

The primary cause of cancer treatment failure is the development of drug resistance. Intrinsic or innate drug resistance exists before the patient is exposed to anticancer drugs, leading to insufficient effectiveness of the treatment. On the other hand, some tumors initially respond to therapy but eventually become resistant over time [1,2]. Malignant cells may either be resistant to a single applied drug (individual resistance) or develop cross-resistance to a wide range of structurally and functionally unrelated agents—a phenomenon known as multidrug resistance (MDR) [3]. This phenomenon is highly significant because most anticancer medications have a narrow therapeutic index, meaning there is a minimal variance between the dosage necessary to generate a therapeutic outcome and the dosage that results in toxicity. Consequently, increasing the dosage after resistance emerges in a clinical setting is useless [4].

Numerous mechanisms contribute to the emergence of MDR. These encompass diminished drug accumulation within the cell, alterations to the detoxification system, modifications of target molecules, the rapid repair of DNA damage, and the prevention of apoptosis [5]. The reduced intracellular accumulation of drugs can be attributed to diminished uptake into the cell and an increased elimination of drugs from the cell. Most chemotherapeutic drugs enter the cell through passive diffusion across the membrane, making changes in the membrane structure capable of affecting drug uptake into the cancer cell. However, the primary reason for reduced accumulation is the effective elimination of chemotherapeutic agents from the cell due to the activity of ATP-binding family (ATP Binding Cassette—ABC) transporters, particularly ABCB1 (P-glycoprotein, MDR1), ABCC1 (MDR protein 1, MRP1), and ABCG2 (breast cancer related protein, BCRP) [6,7].

### 1.1. P-gp Role and Regulation in Cellular Physiology

P-glycoprotein (P-gp) is a well-studied member of the ABC transporter family. This transmembrane protein relies on ATP hydrolysis to actively efflux various substrates from cells. P-gp is involved in a range of physiological processes and is distributed in several tissues, including the liver, intestines, kidneys, placenta, endothelial cells of testicular vessels, and the blood–brain barrier (BBB) [8]. In these tissues, P-gp regulates the cellular uptake rate of xenobiotics, as well as their distribution and excretion, thereby safeguarding vital organs against xenobiotic damage. Moreover, P-gp impacts the effectiveness and bioavailability of medications by modulating their absorption, distribution, metabolism, excretion, and toxicity (ADMET) [9]. In the epithelial linings of the intestines and liver, P-gp has a protective function by actively removing harmful molecules into the bile duct and intestinal lumen [10]. Several studies have also shown that P-gp is involved in lipid homeostasis and cholesterol trafficking [11,12]. In the kidneys, P-gp is specifically localized to the apical surface of the epithelial cells within the proximal tubule. It is postulated that P-gp serves to impede the accumulation of injurious substances in instances of renal injury [13,14]. As a crucial element of the BBB, P-gp is expressed on the apical surface of capillary endothelial cells, where it prevents the entry of toxic substances into the brain [15]. In the testis, P-gp interacts with tight-junction proteins and plays an essential role in the restructuring of the blood–testis barrier during spermatogenesis [16]. P-gp also protects the fetus from maternally transported steroids, toxins and xenobiotics by playing a central role in the placental barrier [17].

P-gp consists of two homologous parts, each containing a transmembrane domain (TMD) with six hydrophobic membrane-spanning α-helices and a cytoplasmic nucleotide-binding domain (NBD) [18]. All 12 α-helices of both TMDs enclose the inner cavity extending from the phospholipid bilayer’s center to the cytoplasm [19]. The cytoplasmic NBDs bind ATP and have ATPase activity. In the inward-facing conformation with separated NBDs, P-gp has numerous drug-binding sites containing hydrophobic and aromatic residues and polar amino acids. These binding pockets allow for the binding of various compounds with different structures and biological activities, including anticancer drugs, antihistamines, calcium channel blockers, antibiotics and many other drugs [20]. The binding of ATP molecules to NBDs leads to their dimerization and switch from the inward-facing conformation to the outward-facing conformation. The change in conformation leads to a rotation of the TMDs and a contraction of the inner cavity, allowing the bound substrates to efflux out of the cell [21]. ATP plays an essential role in stabilizing the outward conformation.

Various transcription factors and signaling pathways regulate the expression level of P-gp. The gene that codes for P-gp, *ABCB1*, is located on chromosome 7 and has 29 exons. The promoter region of the *ABCB1* gene contains an inverse CCAAT element, which interacts with the transcription factors NF-Y and YB-1, as well as a GC element, which interacts with the transcription factors Sp1 and Sp3. These two elements are responsible for the constitutive expression of the *ABCB1* gene. However, the activation of the Ras/MEK/ERK signaling pathway leads to the increased binding of the transcription factors Sp1 and Sp3 to the GC element and the increased transcription of the *ABCB1* gene [22]. Similarly, activating the same signaling pathway leads to phosphorylation and the translocation of the transcription factor YB-1 into the nucleus, increasing the expression of P-gp [23]. Other known inducers of P-gp expression are nuclear factor kappa B (NF-κB) and activator protein-1 (AP-1) [24]. In response to AKT phosphorylation via the JNK pathway, NF-κB translocates to the nucleus and increases the expression of P-gp [25]. Conversely, the tumor suppressor p53 binds directly to the promoter of the *ABCB1* gene and suppresses its expression. Consequently, mutations in the *TP53* gene increase P-gp expression due to the loss of function of the p53 protein [22]. Numerous studies have shown that microRNAs (miRs) such as miR-27a, miR-145 and miR-331-5p decrease the expression of P-gp by interacting with the 3′ untranslated region of *ABCB1* mRNA [26,27]. miR-137 can also reduce the expression of P-gp by modulating the transcription factor YB-1 [28].

### 1.2. P-gp Role in Cancer Therapy and Efforts to Overcome Multidrug Resistance

In addition to its physiological role, P-gp is also involved in establishing and maintaining a resistant phenotype in cancer cells. The upregulation of P-gp in cancer cells is described as an adaptive response to escape chemotherapy-induced cell death. Due to its broad substrate specificity, many anticancer drugs, including anthracyclines, taxanes, vinca alkaloids, epipodophyllotoxins and various tyrosine kinase inhibitors, are substrates for P-gp [29,30]. However, given the ability of P-gp to export a variety of substrates, its enhanced expression in cancer stem cells, and increased mitochondrial ATP production, it has been suggested that P-gp not only exports drugs from cells but also transports cell signaling molecules that contribute to tumorigenesis [31].

Colorectal, renal and hepatocellular carcinomas originate from tissues that initially have elevated P-gp expression and are often characterized by intrinsic resistance [32]. In leukemia, lymphoma and multiple myeloma, P-gp is frequently overexpressed after chemotherapy and cancer recurrence [32]. In acute myeloid leukemia, P-gp is expressed in about 30% of patients at the time of diagnosis and is present in most cases at relapse [33]. Studies have also shown that intrinsic P-gp expression in non-small cell lung cancer (NSCLC) is similar to normal epithelial tissues [34]. However, there is a significant upregulation of P-gp expression during chemotherapy, often associated with a lower survival rate [35].

In brain tumors, capillary endothelial cells express P-gp in 80% of patients, whereas tumor cells express P-gp in 20%. Remarkably, P-gp is not present in the neovasculature of other primary tumors [36]. Both low-grade and high-grade gliomas show P-gp vascular staining. Primary neuroblastomas originating from the adrenal glands show a similar vascular staining pattern as high-grade primary brain tumors [37]. In the study by Demeule et al., P-gp was detected in 60 human brain tumors, including meningiomas, schwannomas and both low- and high-grade gliomas [38]. P-gp is heterogeneously expressed in the membrane and the cytoplasm in glioma cells. Interestingly, P-gp is rarely found in metastatic brain tumors [37,38].

P-gp has served as the primary therapeutic target in various strategies to overcome MDR. Over the years, numerous efforts have been made to incorporate P-gp inhibitors into cancer treatment regimens. Regardless of successful efforts to reverse MDR in cell culture, the shortcoming in clinical application is the lack of specificity and high toxicity [39]. First-generation inhibitors like verapamil, quinidine, and cyclosporine A are compounds already used in the clinic for other indications. These inhibitors were initially identified as P-gp substrates that inhibit P-gp function by competing with other P-gp substrates [40,41]. However, their clinical application is limited due to their high toxicity and low affinity to P-gp. Second-generation inhibitors, designed as analogues of the first-generation inhibitors, exhibit a significantly higher affinity for P-gp [42]. These inhibitors also inhibit CYP3A enzymes, which are crucial for the metabolism of many anticancer drugs. This dual action can potentially alter the pharmacokinetics of anticancer drugs, thus increasing chemotherapeutic toxicity [41]. Subsequently, third-generation inhibitors have been developed with improved specificity for P-gp and a minimal impact on CYP3A enzymes. Important representatives of the third generation of inhibitors are zosuquidar (LY335979), elacridar (GF120918), laniquidar (R101933) and tariquidar (XR9576) [41]. These newer inhibitors are effective at lower doses and achieve optimal efficacy in the nanomolar range. However, clinical trials with tariquidar have raised significant concerns regarding toxicity, and phase III clinical trials were discontinued [43]. In addition, a phase III trial of zosuquidar failed to demonstrate improved outcomes in patients with acute myeloid leukemia [44]. 

Despite the initial promise in preclinical and early clinical trials, the setbacks highlight the challenges of developing effective P-gp inhibitors for clinical use. These results underscore the need to research safer and more-efficient strategies to address MDR in cancer treatment. One notable development is the emergence of natural product drugs as fourth-generation P-gp inhibitors. Plants’ vast chemical diversity provides endless opportunities for discovering new P-gp inhibitors, and their derivatization allows for fine-tuning their performance, especially for natural product drugs that can inhibit P-gp while avoiding being transported by it.

## 2. Modulation of P-gp Activity by Natural Product Drugs

In this review paper, we aim to shed light on several potent classes of natural compounds and their important representatives, which we have been researching for over a decade. These include sesquiterpenoids, especially artemisinin and its derivatives; diterpenoids such as sclareol and its derivatives; and jatrophanes, chalcones, riccardins, and diarylheptanoids (Table 1). Apart from exhibiting strong anticancer activities and selectivity towards cancer cells, these compounds have also demonstrated the ability to affect P-gp and modulate MDR, thus contributing to the ongoing efforts to identify promising candidates for further clinical investigations. Furthermore, we have observed that the chemical derivatization of certain natural compounds discussed herein has led to improved performance against P-gp and enhanced selectivity towards cancer cells.

### 2.1. Sesquiterpenoids

Sesquiterpenoids have demonstrated strong antitumor properties by inhibiting P-gp, suggesting their potential to overcome MDR in cancer therapy. Among them, artemisinin and its derivatives exhibited significant potential in this direction.

#### 2.1.1. Genus *Artemisia*

*Artemisia* is a large, diverse genus of plants belonging to the daisy Asteraceae family, with almost 500 species. Artemisia plants include robust herbaceous varieties and shrubs known for their strong essential oils, which are rich in chemical components. These plants thrive in the temperate climates of the northern and southern hemispheres and generally prefer dry or semi-dry areas. Well-known species are *A. vulgaris* (common mugwort), *A. annua* (sagewort), *A. tridentata* (big sagebrush), *A. dracunculus* (tarragon), *A. absinthium* (wormwood), and *A. abrotanum* (southernwood). Their distinct aroma and bitter taste come from terpenoids and sesquiterpene lactones, which serve as a deterrent against herbivores and may represent an evolutionary advantage. These plants produce small, wind-pollinated flowers [45,46].

*A. annua*, or sweet wormwood, is one member of this genus and often grows near roads, gardens, fields, etc. It is found in Asia and southeast Europe and is moderately distributed in Serbia, mainly in the Vojvodina province [47]. It has been well-known in Chinese traditional medicine for more than 2000 years but became famous in the 1970s, when artemisinin was isolated by Tu Youyou’s group for the first time [48]. Later, its derivatives artesunate, artemether, and dihydroartemisinin were discovered, in addition to their antimalarial activity together with artemisinin. While the efficacy and low toxicity of artemisinin compounds in the treatment of malaria are well-established, they have also been known to exhibit a range of beneficial effects in other capacities. These include antiviral and fungicidal properties, therapeutic effects in nonmalaria parasitic diseases, anti-inflammatory and antiasthma effects, and potential anticancer activity [49].

**Scheme 1:**
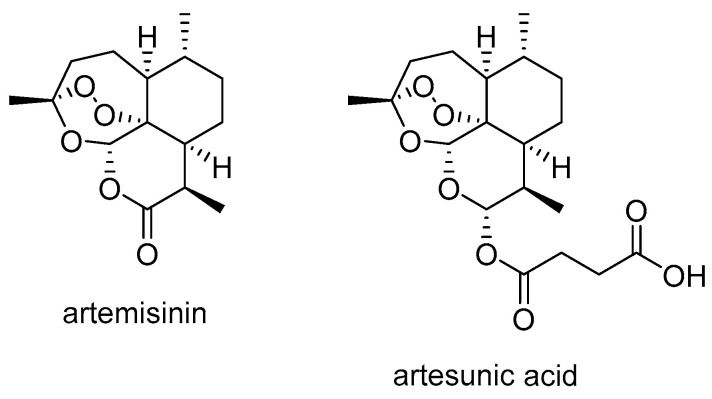
Chemical structures of artemisinin and artesunate.

Artemisinin (Scheme 1), an antimalarial sesquiterpene extracted from *A. annua* [50], and its derivatives have shown significant anticancer activity, including the ability to overcome P-gp-mediated MDR in cancer treatment. While some derivatives are P-gp substrates, many also act as P-gp inhibitors, suggesting that they have the potential to reverse MDR and support the development of new cancer therapies [51].

Artesunate (artesunic acid) (Scheme 1) is a semi-synthetic derivative of artemisinin that has attracted considerable attention in cancer research over the last two decades due to its strong antitumor effect. Studies have shown that artesunate and its derivatives not only inhibit tumor growth but also overcome the MDR by inhibiting P-gp activity, thus increasing the efficacy of conventional chemotherapeutic agents.

In a recent study, artesunate was found to increase the cytotoxicity of doxorubicin in doxorubicin-resistant K562 leukemia cells (K562/ADR) [52]. Artesunate increased the efficacy of doxorubicin by inhibiting glycolysis. In addition, artesunate decreased the expression of the *MDR1* and *ABCG2* genes and inhibited the activities of P-gp and ABCG2 in K562/ADR cells. Artesunate also promoted apoptosis and decreased cell viability.

The promising activity and facile derivatization of the carboxyl group make artesunate a good candidate for transformation into derivatives (e.g., esters and amides) with better biological activity.

**Scheme 2:**
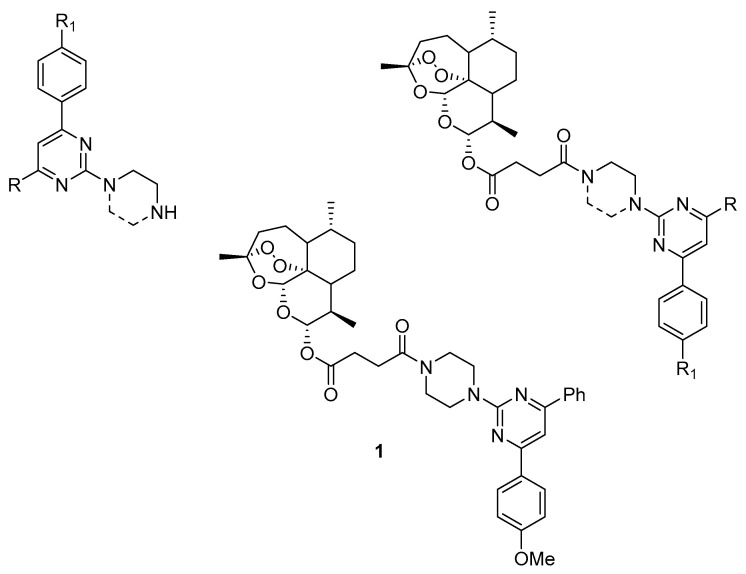
Chemical structures of 4-aryl-2-aminopyrimidines and new artesunate-based hybrids.

A recent study reported the synthesis of 17 hybrid molecules combining artesunate, a derivative of artemisinin, with synthetic 4-aryl-2-aminopyrimidines (Scheme 2) [53]. These new compounds were designed to improve the parent molecules’ cytotoxic properties, activity and selectivity. The hybrid molecules, with an ethylenediamine linker, a piperazine linker, and their precursors, including the pyrimidine derivatives artemisinin and artesunate, were tested against susceptible and MDR human non-small cell lung carcinoma cells. The hybrid molecules with the piperazine linker showed high selectivity against cancer cells. These hybrids had a similar anticancer effect to artesunate but exhibited significantly improved selectivity against cancer cells. The hybrid compounds with the piperazine linker bypassed the MDR phenotype, inhibited P-gp activity and increased MDR cancer cells’ sensitivity to doxorubicin. P-gp inhibition by several piperazine-based hybrid compounds was more effective than that by artesunate. Compound **1** (Scheme 2) proved to be the most effective anticancer agent in both sensitive and MDR lung cancer cells.

**Scheme 3:**
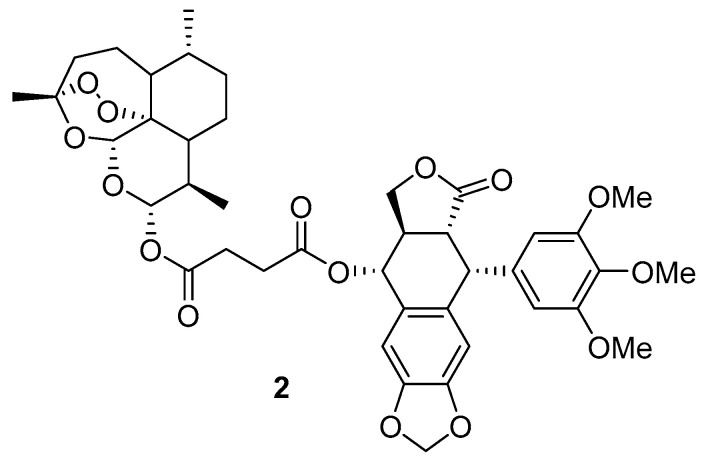
Chemical structure of artesunate-podophyllotoxin conjugate.

Artesunate was also found to block rhodamine 123 transport in the Caco-2 cell model and decrease P-gp-mediated digoxin transport [54]. A new artesunate-podophyllotoxin conjugate **2** (Scheme 3) was found to have strong cytotoxicity against cancer cell lines and reduced drug resistance [55]. The conjugate decreased P-gp levels in P-gp-overexpressing K562/ADR cells. It also disrupted the microtubule network, caused G_2_/M cell cycle arrest in K562/ADR cells and induced apoptosis.

Natural bicyclic sesquiterpenes, β-caryophyllene (BCP) and β-caryophyllene oxide (BCPO), are present in a large number of plants worldwide, including the genus *Artemisia,* and are often found in essential oils. BCP is a plant compound, a member of the bicyclic sesquiterpenes. In nature, it mainly occurs as trans-caryophyllene ((E)-BCP) mixed with small amounts of its isomers, (Z)-β-caryophyllene (iso-caryophyllene) and α-humulene (α-caryophyllene), as well as its oxidation derivative BCPO.

**Scheme 4:**
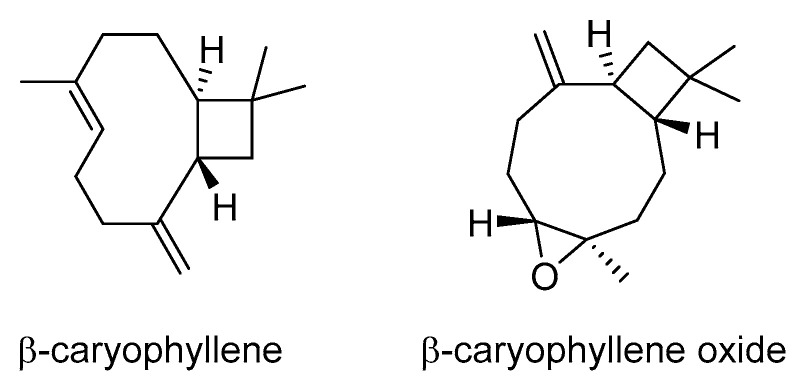
Chemical structures of β-caryophyllene and β-caryophyllene oxide.

BCP and BCPO (Scheme 4) have been shown to enhance the effectiveness of low-dose doxorubicin in treating human hepatoma HepG2 cells [56]. These sesquiterpenes not only increased the intracellular accumulation of doxorubicin and rhodamine 123 but also interfered with the function of P-gp. Molecular docking studies revealed that BCP has a strong binding affinity to P-gp. 

#### 2.1.2. Genus *Curcuma*

*Curcuma* is a genus of plants in the Zingiberaceae family that contains more than 130 species that are native to southeast Asia, southern China, the Indian subcontinent, New Guinea and northern Australia. 

*Curcuma longa* is one of the best-known species within its genus and belongs to the Zingiberaceae family. This perennial herbaceous plant grows from rhizomes and is native to the Indian subcontinent and southeast Asia. It thrives in regions with temperatures between 20 and 30 °C and requires a lot of rainfall each year for optimal growth [57]. 

**Scheme 5:**
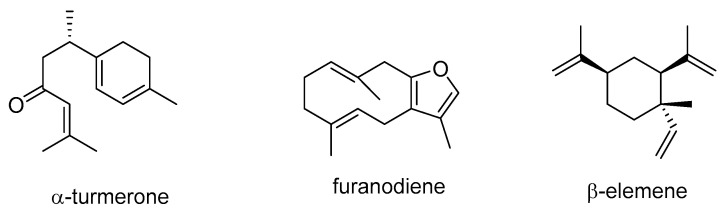
Chemical structures of α-turmerone, furanodiene, and β-elemene.

Turmerones, a group of sesquiterpenoids found in *Curcuma longa*, were investigated for their impact on the absorption and transport of curcumin in human colorectal adenocarcinoma Caco-2 cells [58]. The study revealed that the presence of α- and aromatic turmerones significantly enhanced the transport of curcumin into Caco-2 cells. Additionally, α-turmerone (Scheme 5) was found to inhibit the activity of P-gp, potentially explaining the increased curcumin uptake. Conversely, aromatic turmerone increased P-gp mRNA expression. 

Furanodiene (Scheme 5), a precursor of furan-containing sesquiterpenoids isolated from *Rhizoma curcumae*, enhanced the anticancer potential of 5-FU, reversing resistance to cisplatin and doxorubicin in xenotransplanted zebrafish cancer models. These effects could be related to its ability to inhibit angiogenesis, induce reactive oxygen species (ROS) production, cause DNA strand breaks, and promote apoptosis by blocking P-gp efflux transport [59]. β-Elemene (Scheme 5), a sesquiterpenoid extracted from *Rhizoma curcumae*, affects cellular signaling pathways involved in cell survival and apoptosis. It is a promising candidate for cancer treatment due to its efficacy, safety, and synergistic effects with other substances. A study by Zhang et al. suggested that β-elemene could be used alongside conventional P-gp substrate chemotherapeutics to counteract MDR in leukemia treatment [60]. Additionally, β-elemene may act as an anti-metastatic agent when combined with doxorubicin in the treatment of MDR gastric cancer. The mechanisms underlying these effects include β-elemene’s ability to enhance the efficacy of doxorubicin by suppressing P-gp expression, increasing intracellular doxorubicin concentrations, and modulating signaling pathways related to Akt phosphorylation, E3 ubiquitin ligases, and miR-1323/Cbl-b/EGFR [60,61]. Tang et al. found that β-elemene caused the reversal of doxorubicin resistance in MCF-7 cells by decreasing ABC transporter function and ABC gene and protein expression [62].

#### 2.1.3. Genus *Ferula*

*Ferula* species belong to the Apiaceae family. The genus *Ferula*, the third-largest genus of the Apiaceae (alt. Umbelliferae) family, is composed of approximately 180 species distributed in central and southwest Asia, the Far East, north India and the Mediterranean. Fifteen of them are endemic to Iran, nine to Turkey, seven to China and one species to Italy, and the rest are indigenous entities of several other countries [63]. Plants of this genus are rich sources of biologically active natural products such as sesquiterpene coumarins, sulfur-containing compounds and sesquiterpene lactones. 

**Scheme 6:**
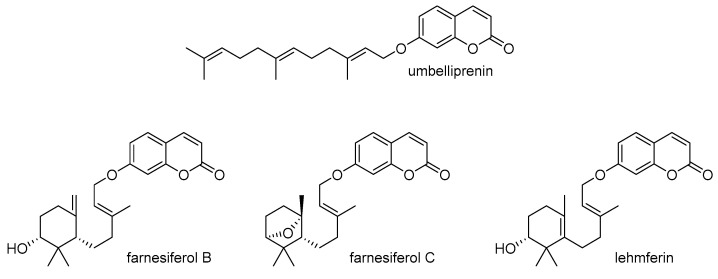
Chemical structures of umbelliprenin, farnesiferol B, farnesiferol C, and lehmferin.

In a study by Kasaian et al., fifteen sesquiterpene coumarins from different *Ferula* species were examined for their potential to reverse MDR [64]. When used at very low concentrations, certain sesquiterpene coumarins such as umbelliprenin, farnesiferol B, farnesiferol C, and lehmferin (Scheme 6) significantly enhanced the cytotoxicity of doxorubicin in P-gp-overexpressing MCF-7/ADR cells. These compounds also led to an increased intracellular accumulation of rhodamine 123 in the treated cells. Notably, the ring-opened drimane-type sesquiterpene coumarins, particularly farnesiferol B, farnesiferol C, and lehmferin, demonstrated the strongest inhibitory effect on P-gp efflux, suggesting their potential for further structural modifications to improve their MDR-reversal properties.

#### 2.1.4. Genus *Inula*

The *Inula* genus comprises approximately 120 species of flowering plants within the Asteraceae family, originating from Europe, Asia, and Africa. There are 35 species of this genus in Europe and 11 in Serbia. *I. helenium* can be found in central and southeast Europe and central Asia. It is widely distributed in Serbia. *I. oculus-christi* L. can be found on the Balkan Peninsula and in surrounding countries, including central and south Russia, the Caucasus Mountains, Armenia and Anatolia. It is also widely distributed in Serbia. *I. Britannica* L. grows in central and south Europe and Iran. Its habitat is in central Serbia, near roads, forests, and creeks [65].

**Scheme 7:**
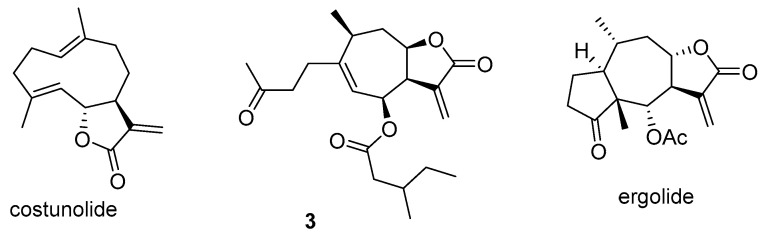
Chemical structures of costunolide, sesquiterpenoid **3**, and ergolide.

Costunolide (Scheme 7), derived from *I. helenium* L., demonstrated an antiproliferative effect on various cancer cells and significantly enhanced the antiproliferative efficacy of doxorubicin in drug-resistant cell lines by blocking the PI3K/AKT signaling pathway and reducing P-gp expression [66,67]. Ergolide (Scheme 7), isolated from *I. oculus-christi* L., exhibited strong synergistic effects with vincristine against acute lymphoblastic leukemia cells, indicating its potential to overcome P-gp-mediated resistance [68]. Compound **3** (Scheme 7) from *I. japonica* was found to inhibit the expression of drug-resistance proteins such as ABCC1, ABCG2, and P-gp. This suggests a potential mechanism for reversing MDR and improving chemotherapy sensitivity in the paclitaxel-resistant human non-small cell lung cancer cell line A549/PTX [69]. In another study, sesquiterpene lactones isolated from *I. britannica* from Serbia were screened for cytotoxic activity on four different human cancer cell lines and their multidrug-resistant counterparts, as well as on normal human keratinocytes, and they showed similar cytotoxic activity toward drug-sensitive and drug-resistant cancer cell lines [65].

#### 2.1.5. Genus *Petasites*

*Petasites* belongs to the Asteraceae family, which is known for its flowering plants. These perennial plants have robust underground rhizomes that spread horizontally, and during their growth phase, they form large, rhubarb-like leaves. Most *Petasites* species originate from regions in Asia or southern Europe [70]. *Petasites formosanus* Kitamura is a perennial herb and the only indigenous *Petasites* species in Taiwan [71].

**Scheme 8:**
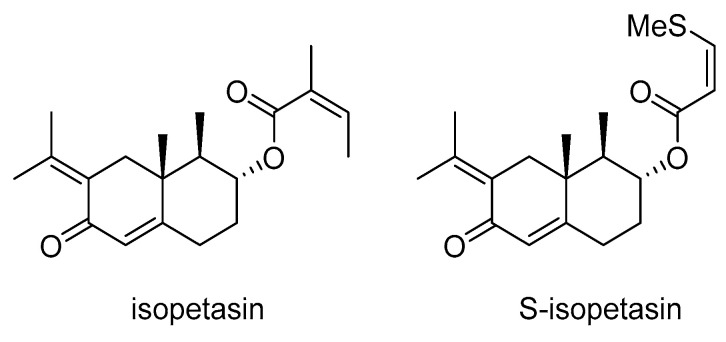
Chemical structures of isopetasin and S-isopetasin.

Two sesquiterpenes, isopetasin and S-isopetasin (Scheme 8), found in *Petasites formosanus*, have a dual function in cancer therapy. They inhibit P-gp and also have cytotoxic effects [72]. A study by Abdelfatah et al. demonstrated that these compounds bind with high affinity to key amino acids of the P-gp transporter. This binding leads to increased mitochondrial workload, ATP production, ROS formation, and ultimately cell death in the MDA-MB-231 cell line [72].

#### 2.1.6. Genus *Celastrus*

The genus *Celastrus* (Celastraceae) comprises about 30 species worldwide, consisting of deciduous or evergreen shrubs with cylindrical branches, typically reaching a height of 1–10 m. These plants have a long history in Asia, where they are used in traditional medicine for a variety of ailments, including arthritis, rheumatoid arthritis, cognitive disorders, insomnia, edema, epilepsy, joint and muscle pain [73].

Callies et al. created a natural product-based library based on the dihydro-β-agarofurans, previously identified in *Celastrus vulcanicola*. The library was screened for its ability to inhibit P-gp-mediated daunomycin efflux in MDR cells [74]. Several analogs showed higher potency than the lead compound and a first-generation P-gp modulator, verapamil, indicating their potential as effective modulators of P-gp-mediated MDR in cancer cells.

### 2.2. Diterpenoids

Diterpenoids are wide range of plant secondary metabolites formed from four isoprene units found in large number of species. In this section, we will discuss diterpenoids found in some plant species from Serbia and neighboring countries with the potential to inhibit the growth of cancer cells and overcome drug-resistance mechanisms mediated by P-gp.

#### 2.2.1. Genus *Salvia*

Diterpenoids are wide range of plant secondary metabolites formed from four isoprene units found in a large number of species. *Salvia* is the largest genus of the Lamiaceae family and includes near 900 species. *S. sclarea* grows mainly in the Mediterranean region, from Caucasus, Iran and Syria to southern regions of France and northern Africa. In Serbia, it can be found in the eastern and southeastern parts of the country [75]. *S. officinalis* habitats are also in the Mediterranean region, from Spain to the northern Balkan regions, eastern Asia, and northern Syria. In Serbia, it is widely distributed, especially in Artemisio-Salvietum officinalis association [76]. It is cultivated widely across the whole of Europe and North America.

**Scheme 9:**
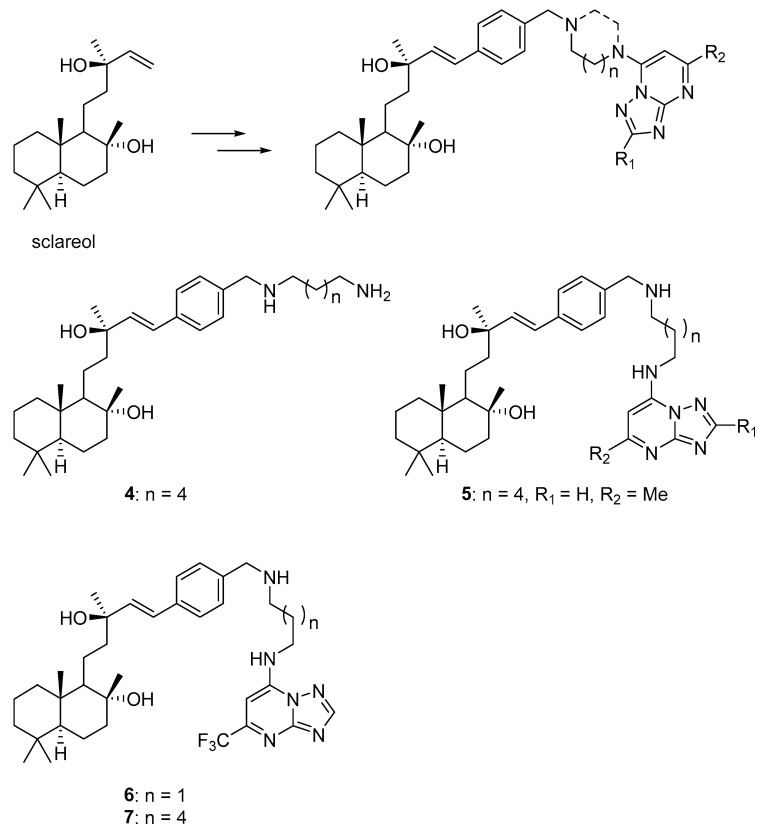
Chemical structures of sclareol, its derivatives and hybrids with 1,2,4-triazolo[1,5-a]pyrimidines.

Sclareol (Scheme 9), a natural labdane diterpene, has been extensively studied for its anticancer properties and its ability to reverse resistance to various drugs. Isolated from *Salvia sclarea* and *Salvia officinalis*, sclareol has shown promising results in preclinical studies as a cytotoxic agent against cancer cells and, more recently, as a P-gp modulator. These findings suggest its potential as a therapeutic agent in cancer treatment.

Sclareol has shown potential in reducing cell viability and increasing apoptosis in MCF-7 breast cancer cells [77]. Combined with cyclophosphamide, sclareol enhanced these anticancer effects. Sclareol increased the expression of p53 and BAX while downregulating Bcl-2. Docking studies revealed an interaction between sclareol and STAT3 and inhibited the role of IL-6 in the modulation of apoptosis-associated genes. Additionally, sclareol treatment in the MKN-45 gastric cancer cell line resulted in a significant reduction in *MDR1* gene expression and P-gp levels at lower doses [78]. Sclareol also reduced the viability of human small-cell lung cancer cells [79] and inhibited tumor weight and volume in a xenotransplanted-tumor mouse model.

Sclareol also demonstrated the potential to reverse cisplatin resistance in non-small cell lung cancer cells [80]. In HeLa cells, sclareol inhibited cell proliferation and induced apoptosis [81]. By encapsulating doxorubicin and sclareol together, researchers found that a nanostructured lipid carrier (NLC-DOX-SC) is a viable alternative to the DOX:SC combination with fewer side effects [82].

Sclareol was found to increase the accumulation of doxorubicin in glioblastoma cells, regardless of the expression and activity of P-gp. This effect was observed in all the glioblastoma cell lines studied, including U87, U87-TxR, and U251 [83]. The most significant impact was seen in the MDR cell line U87-TxR, which showed an increase in accumulation of over 50%. These findings prompted the development of new hybrid compounds of sclareol and doxorubicin, which exhibited greater selectivity towards cancer cells and displayed reduced resistance compared to doxorubicin in glioblastoma cells [83].

Dimas and co-authors published several studies regarding the cytotoxic activity of sclareol. They demonstrated that sclareol induces apoptosis in human leukemic cell lines by downregulating the expression of proto-oncogene c-myc without affecting the expression of the anti-apoptotic protein, Bcl-2 [84,85], and in human colon tumor cells (HCT116) in vitro by activating both the mitochondrial pathway and the death receptor pathway [86]. Additionally, the same group reported that sclareol enhances the activity of known anticancer drugs such as doxorubicin, etoposide, and cisplatin against human breast cancer cells [87], in addition to inducing apoptosis in human tumor cell lines and suppressing tumor growth in vivo via a p53-independent mechanism of action [88].

In a recent study, 24 hybrid molecules were synthesized by combining naturally occurring sclareol and synthetic 1,2,4-triazolo[1,5-*a*]pyrimidines to improve cytotoxic properties, activity, and selectivity [89]. Due to the chemoselectivity of the allyl alcohol functional group, the tertiary and tertiary allylic hydroxyl groups of sclareol were preserved during the derivatization, which is essential for the desired biological activity. The concentration-dependent cytotoxicity of these molecules was examined in MDR U87-TxR cells with increased P-gp expression and normal lung fibroblasts (MRC-5). Two hybrid compounds were active in the nanomolar range, and seven compounds were more selective towards glioblastoma cells than sclareol. Additionally, sclareol and several derivatives showed collateral sensitivity. Furthermore, hybrids **5**, **6**, and **7** (Scheme 9) reduced P-gp activity similarly to tariquidar. Specifically, compound **5** and its precursor **4** (Scheme 9) affected various cellular processes, including cell cycle, cell death, mitochondrial membrane potential, and ROS/RNS levels in glioblastoma cells. The sensitivity of MDR glioblastoma cells was linked to the modulation of oxidative stress and mitochondrial inhibition.

#### 2.2.2. Genus *Euphorbia*

The genus *Euphorbia*, also known as spurge, is part of the Euphorbiaceae family and comprises more than 2000 species of annual, biennial, or perennial flowering plants. It is recognized as one of the largest and most diverse genera within the plant kingdom. Studies on the phytochemical composition of these plants have revealed the presence of a wide range of secondary metabolites, including terpenes, glycerols, steroids, cerebrosides, and phenolic compounds. These compounds have been found to exhibit various beneficial activities, such as antiproliferative, cytotoxic, antiviral, antimicrobial, anticancer, and anti-inflammatory properties [90,91]. Furthermore, the genus *Euphorbia* is abundant in jatrophanes, which are macrocyclic diterpenes characterized by a unique basic structure and a flexible twelve-membered ring.

Over the past two decades, significant research has focused on the effects of jatrophane diterpenoids on P-gp modulation, resulting in a comprehensive understanding of this area. Numerous jatrophane compounds have emerged as strong P-gp inhibitors. The following section will explore the plant sources of jatrophane diterpenes, extending beyond Serbia and its adjacent regions to include other areas, due to their notable efficacy as P-gp inhibitors.

##### *E. nicaeensis* All.

*E. nicaeensis* is a hardy perennial plant characteristic for the Mediterranean region, also found in Serbia, Romania, and Bulgaria. It grows in dry regions in Serbia such as Deliblato Sand in the Vojvodina province.

**Scheme 10:**
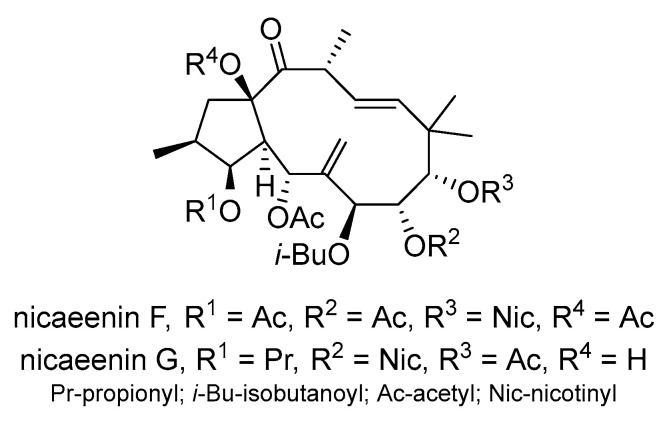
Chemical structures of nicaeenin F and G.

Seven newly identified jatrophane diterpenoids named nicaeenin A–G were discovered from the latex of *E. nicaeensis* in Serbia, in addition to eight known jatrophane diterpenoids—the euphodendrophanes A–C, F, N, O, Q, and S [92]. Most of the newly identified jatrophanes showed significant potential to inhibit P-gp activity in the two MDR cancer cell lines NCI-H460/R and DLD1-TxR. Among them, nicaeenin F and nicaeenin G (Scheme 10) showed the strongest inhibitory effects. Additionally, nicaeenin G significantly increased the sensitivity of NCI-H460/R cells to doxorubicin compared to Dex-verapamil.

**Scheme 11:**
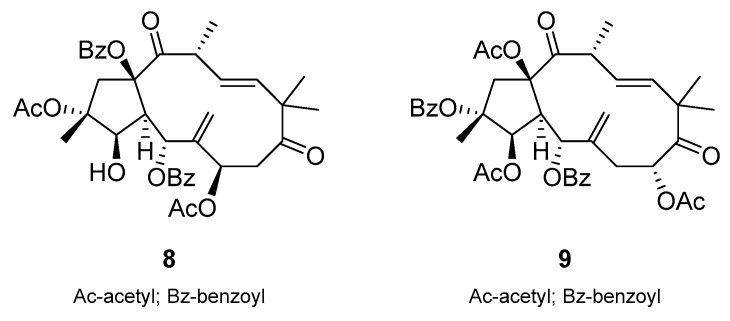
Chemical structures of jatrophane diterpenoids **8** and **9**.

Another study by Krstić et al. reported that jatrophane diterpenoids **8** and **9** (Scheme 11)**,** isolated from the roots of *E. nicaeensis*, effectively inhibited P-gp in MDR cells of non-small cell lung cancer and colorectal carcinoma [93]. The compounds outperformed the potential of the P-gp inhibitor tariquidar in MDR colorectal carcinoma and glioblastoma cells and served as sensitizing agents that were able to reduce the required drug concentrations for P-gp substrate drugs. Jatrophane **1** also inhibited the cell growth of non-small cell lung cancer and glioblastoma cells, suggesting significant anticancer properties.

##### *E. dendroides* L.

*E. denroides* L. is a perennial semisucculent bush with a height up to 3 m. It grows in hilly coastal areas of the Mediterranean, mainly Montenegro, Greece and Albania, on rocky surfaces [94]. This species is well-known for its use as a biomass for biofuel production [95], but it has not been extensively chemically investigated.

**Scheme 12:**
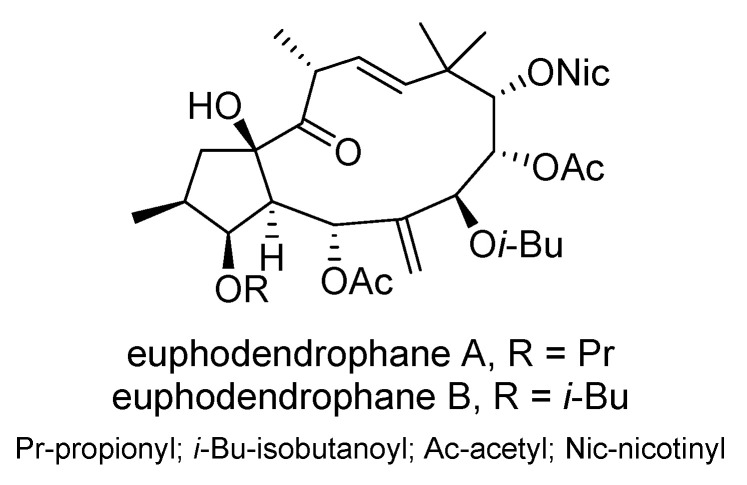
Chemical structures of euphodendrophane A and B.

Six new jatrophane diterpenoids extracted from the Montenegrin spurge *E. dendroides* were found to inhibit the growth of four human cancer cell lines: NCI-H460, NCI-H460/R, DLD1, and U87 [96]. The most potent compounds, euphodendrophane B and the closely related euphodendrophane A (Scheme 12), were tested for their interactions with paclitaxel and doxorubicin, particularly in the MDR non-small lung carcinoma cell line NCI-H460/R. Both compounds demonstrated strong potential to reverse drug resistance by inhibiting P-gp transport, effectively reversing resistance to paclitaxel in NCI-H460/R cells. Additionally, these jatrophanes improved the sensitivity of NCI-H460/R cells to doxorubicin.

**Scheme 13:**
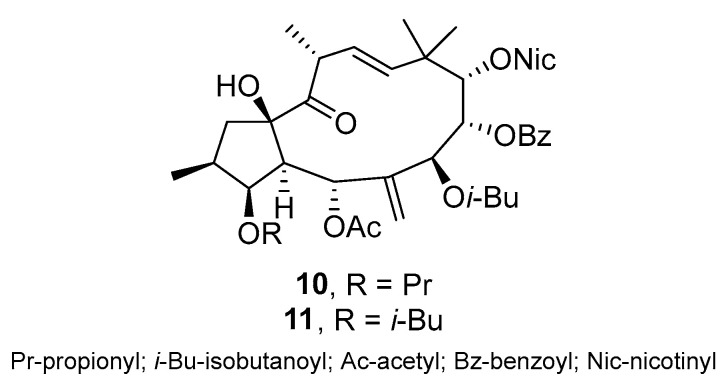
Chemical structures of jatrophane diterpenoids **10** and **11**.

In a study by Jadranin et al., thirteen jatrophane diterpenoids were isolated from the latex of *E.dendroides* [97]. The inhibitory effect of these jatrophane diterpenoids on P-gp was investigated. In particular, compounds **10** and **11** (Scheme 13) showed strong P-gp inhibition, outperforming R(+)-verapamil and tariquidar in the colorectal carcinoma MDR DLD1-TxR cells.

##### *E. esula* L.

*E. esula* L. (leafy spurge) is a species with a worldwide distribution, from Scandinavia to the Balkan Peninsula (mostly in Serbia), from Spain to mid Russia, and in mid Asia, Iran, Siberia, and east Asia. In Serbia, it can be found in the Fruška Gora Mountains and Gledić Mountains [94].

**Scheme 14:**
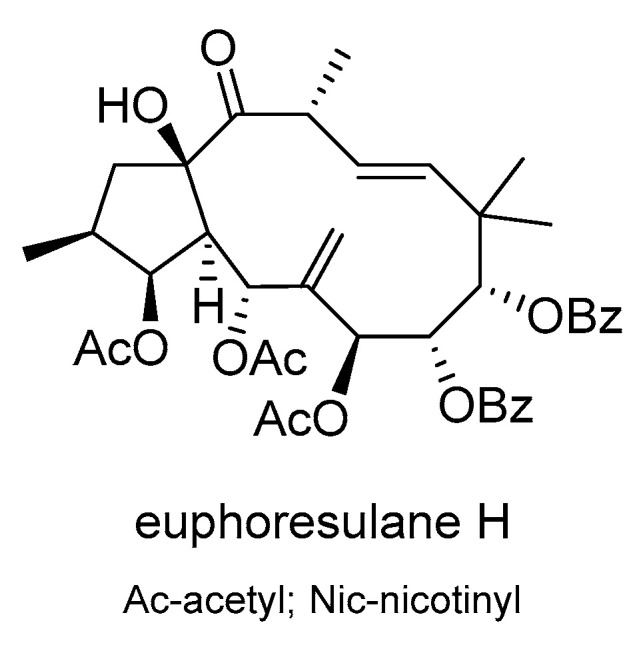
Chemical structures of euphoresulan H.

Thirteen new jatrophane diterpenoids, euphoresulanes A-M, and seven known ones were isolated from *E. esula* [98]. These compounds were assessed for their ability to reverse MDR in P-gp-overexpressing HepG2/ADR hepatocellular carcinoma cells. Euphoresulan H (Scheme 14) proved to be the most potent MDR modulator, increasing the efficacy of doxorubicin by 33-fold.

##### *E. helioscopia* L.

*E. helioscopia* L. is a widely distributed *Euphorbia* species. It can be found across the whole of Europe and in northern Africa and east Asia, but also in other parts of Asia. It is abundant in all regions of Serbia [99].

**Scheme 15:**
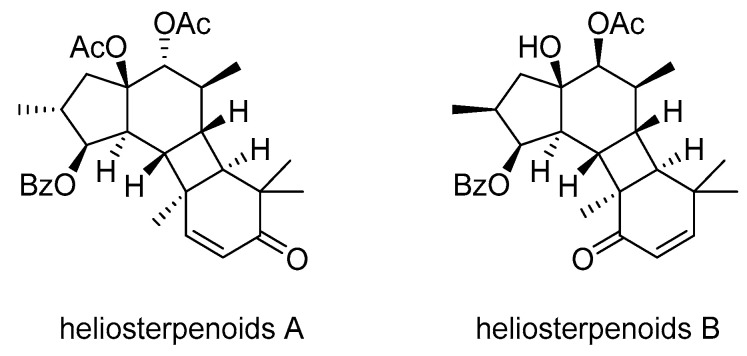
Chemical structures of heliosterpenoids A and B.

Heliosterpenoids A and B (Scheme 15), two novel jatrophane-derived diterpenoid esters with a unique 5/6/4/6-fused tetracyclic ring structure, were isolated from *E. helioscopia* [100]. Both compounds exhibited a strong inhibition of P-gp, with heliosterpenoid A also showing cytotoxicity against the breast cancer cell line MDA-MB-231.

##### *E. sororia* A. Schrenk

**Scheme 16:**
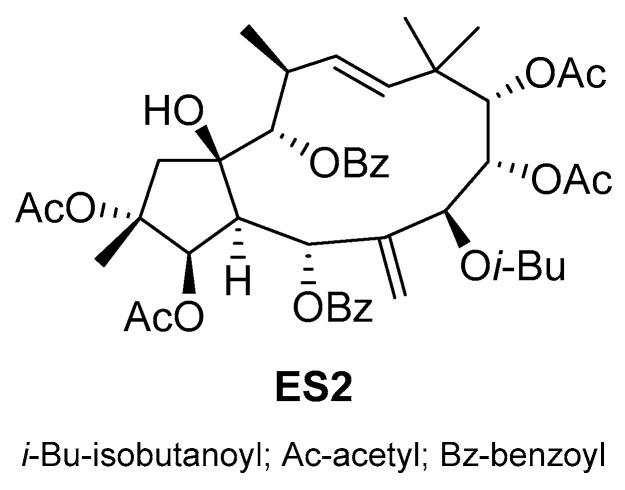
Chemical structure of jatrophane diterpenoid ES2.

ES2 (Scheme 16), a novel jatrophane diterpenoid ester derived from the fruit of *E. sororia*, a traditional Uighur medicinal plant in China, was evaluated for its potential in reversing P-gp-mediated MDR [101]. ES2 showed minimal cytotoxicity against both P-gp-overexpressing MDR cells and their non-resistant counterparts but sensitized MDR cells and P-gp-transfected HEK293 cells to chemotherapeutics known to be P-gp substrates. The reversal effect of ES2 was primarily due to the inhibition of the efflux function of P-gp. Additionally, ES2 stimulated the ATPase activity of P-gp in a concentration-dependent manner without altering P-gp expression. Molecular docking analyses revealed the binding affinity of ES2 to the drug-binding site of the P-gp transporter. ES2 significantly enhanced the antitumor efficacy of vinorelbine against KBv200 cell xenografts in nude mice.

**Scheme 17:**
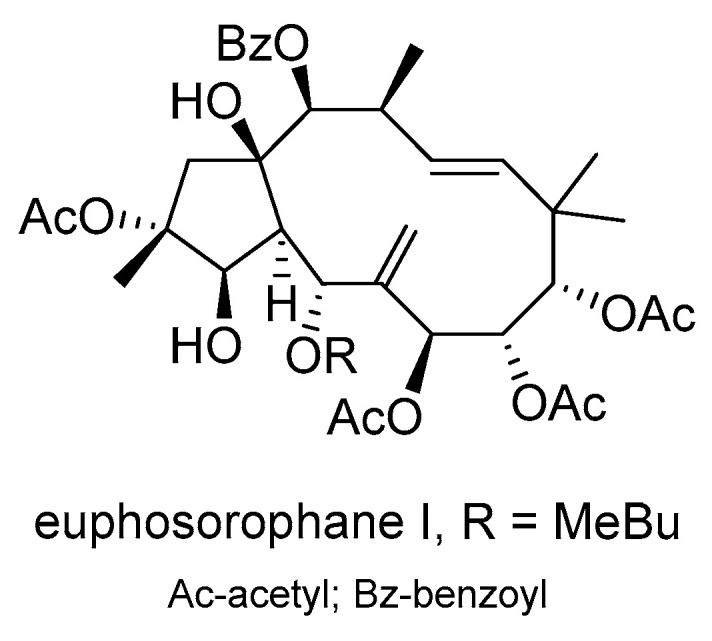
Chemical structure of euphosporophane I.

Eight new jatrophane diterpenoids were found in the fructus of *E. sororia*, along with fourteen known ones [102]. These compounds displayed lower cytotoxicity and showed potential in reversing MDR in doxorubicin-resistant human breast cancer MCF-7/ADR cells. Particularly, euphosorophane I (Scheme 17) demonstrated high efficacy in reversing P-gp-mediated resistance to doxorubicin. It increased the intracellular accumulation of rhodamine 123 and doxorubicin in drug-resistant cells, inhibited P-gp-dependent rhodamine 123 efflux, stimulated P-gp ATPase activity, and inhibited doxorubicin transport. Euphosorophane I acted by activating ATPase rather than upregulating P-gp expression, resulting in a better reversal of MDR. Molecular docking analysis confirmed its binding to the drug-binding site of P-gp.

**Scheme 18:**
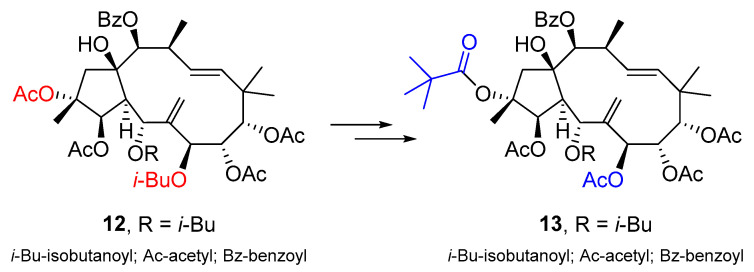
Chemical structures of jatrophane diterpenoid **12** and corresponding derivative **13**.

Maimaitijiang et al. conducted a study focusing on the development of novel modulators targeting P-gp to combat MDR in cancer treatment [103]. They modified compound **12** (Scheme 18), a natural jatrophane from *E. sororia*, to produce nine derivatives that exhibited enhanced MDR-reversal activity in P-gp-overexpressing MCF-7/ADR cells compared to verapamil. The most effective compound, **13** (Scheme 18), abolished P-gp-mediated resistance to doxorubicin, with low cytotoxicity and a high therapeutic index. Compound **13** not only increased the accumulation of doxorubicin and rhodamine123 in MCF-7/ADR cells but also stimulated P-gp ATPase activity, ultimately increasing doxorubicin sensitivity. The mechanism of action for the reversal activity of compound **13** was through the stimulation of P-gp ATPase activity and not through the direct inhibition of P-gp protein expression. Molecular docking studies confirmed its high binding affinity with the doxorubicin-recognition site on P-gp.

##### *E. glomerulans* Prokh.

**Scheme 19:**
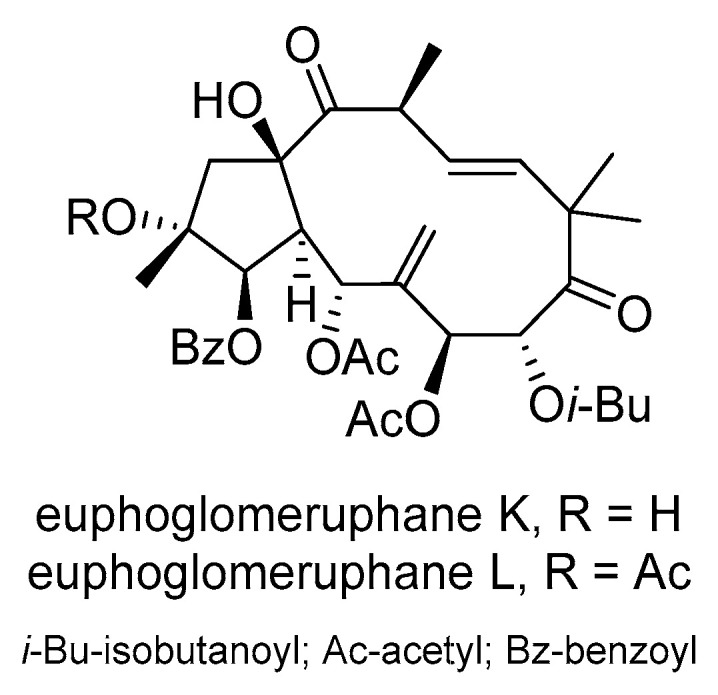
Chemical structures of euphoglomueruphane K and L.

*E. glomerulans* Prokh. is distributed in northwestern China and the five countries that make up central Asia. A cytotoxic evaluation of jatrophane diterpenoids isolated from the acetone extracts of *E. glomerulans* on MCF-7/ADR found that several compounds were able to reverse MDR [104]. Among these, euphoglomeruphane K and L (Scheme 19) showed superior MDR-reversal activity compared to the positive control verapamil.

**Scheme 20:**
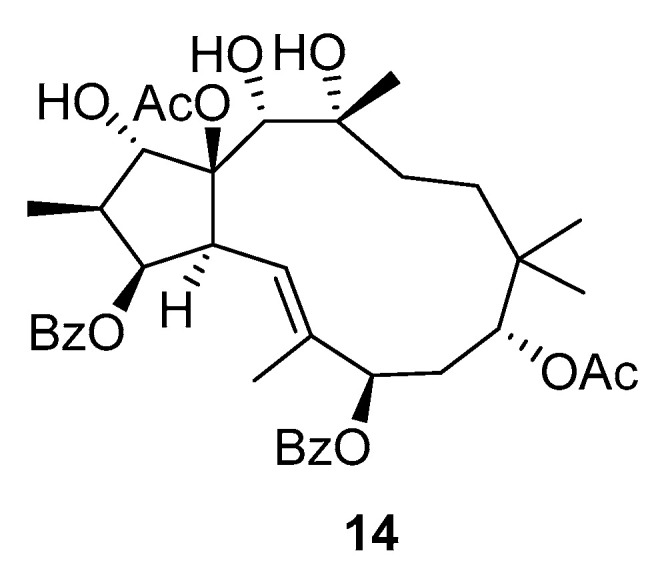
Chemical structures of jatrophane diterpenoid **14**.

Another example of biologically active jatrophane ditrpenoids are those from *Pedilanthus tithymaloides* (*Euphorbia tithymaloides*). It is native to tropical and subtropical North America and Central America. Thirteen jatrophane diterpenoids, including eight newly discovered compounds, were isolated from *P. tithymaloides* [105]. Modifications of these compounds led to 22 new derivatives, forming a library for screening P-gp-dependent MDR modulators. Compound **14** (Scheme 20) in particular showed exceptional stability and promising antitumor effects, making it a leading candidate for the development of new MDR-reversal agents. The structure–activity relationship (SAR) results for the jatrophane diterpenoids strongly indicate that the position and nature of ester functional group have a key role in the bioactivity of this class of compounds.

### 2.3. Chalcones

Chalcones are a group of plant polyphenol compounds that belong to the flavonoid family. Numerous studies have highlighted the potential of natural chalcones to counteract resistance to conventional chemotherapeutics by targeting key multidrug efflux transporters in cancer cells. The ability of naturally occurring chalcones to inhibit transporters such as P-gp has generated great interest in recent years in their use as chemosensitizers to improve drug efficacy and overcome clinical drug resistance in cancer treatment.

#### 2.3.1. Genus *Piper*

The genus *Piper* belongs to the Piperaceae family. It contains up to 2000 species of shrubs, herbs, and lianas, many of which are dominant species in their native habitat. The diversification of this taxon is of interest to understanding the evolution of plants. Piper species have a pantropical distribution and are most commonly found in the understory of lowland tropical forests, but they can also occur in clearings and in higher-elevation life zones such as cloud forests [106].

*Piper methysticum* Forster f. (Piperaceae) is a tropical shrub belonging to the Piperaceae family. The common names for *P. methysticum* extract include ava, ava pepper, awa, intoxicating pepper, kava, kava kava, kava pepper, kawa, kawa kawa, kew, rauschpfeffer, sakau, tonga, wur zelstock, and yangona. The active principles of Kava Kava rootstock are mostly contained in the lipid-soluble resin, which is generally grouped into categories: arylethylene-apyrones, chalcones and other flavanones, and conjugated diene ketones [107].

**Scheme 21:**
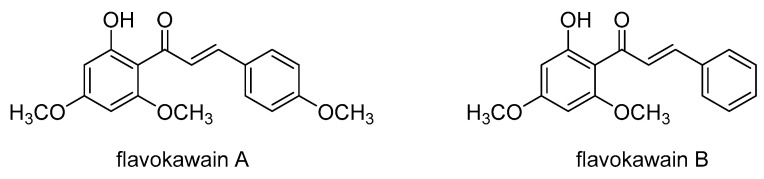
Chemical structures of flavokawains A and B.

Flavokawain A (Scheme 21), a chalcone compound found in kava, *Piper methysticum*, has strong anticancer properties. A study by Li et al. investigated the effect of flavokawain A on the viability of paclitaxel-resistant A549/T lung cancer cells and its potential hepatotoxicity in normal liver epithelial cells [108]. The study found that flavokawain A effectively inhibited the proliferation of A549/T cells and induced apoptosis in a dose-dependent manner. Furthermore, flavokawain A showed no hepatotoxic effects on liver epithelial cells. Treatment with flavokawain A reduced the P-gp expression threefold after 24 h by inhibiting the PI3K/Akt signaling pathway, suggesting that flavokawain A may be a promising treatment option for paclitaxel-resistant lung cancer. Another study showed that flavokawain B (Scheme 21), another chalcone from the kava plant, exhibited cytotoxic and antiproliferative effects on doxorubicin-resistant human colorectal adenocarcinoma cells (LoVo/Dx), although the exact mechanism on the ABCB1 transporter is still unclear [109].

#### 2.3.2. Genus *Helichrysum*

The genus *Helichrysum* comprises about 600 species of flowering plants within the family Asteraceae, commonly known as the sunflower family. These plants are distributed throughout Africa; they are particularly common in South Africa, with 244 species, as well as in Madagascar, Australasia and Eurasia [110].

The species *Helichrysum zivojinii* Černjavski and Soška is an endemic wild-growing plant in the Republic of North Macedonia at Galičica Mountain [111]. Our recent studies of the extracts obtained from the aerial parts of *H. zivojinii* revealed their specific cytotoxic action towards malignant cell lines in comparison with healthy immunocompetent peripheral blood mononuclear cells (PBMCs) [112] and their potential ability to affect steps in the progression of malignant tumors [111].

**Scheme 22:**
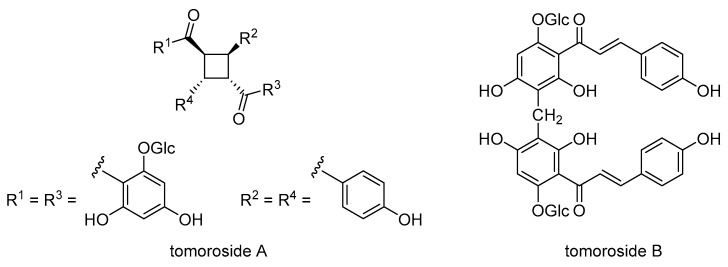
Chemical structures of tomoroside A and B.

Chalcone dimers tomoroside A and tomoroside B (Scheme 22) were isolated together from the aerial parts of *H. zivojinii* [113]. Tomoroside A inhibited the expression of Topo IIα and HIF-1α and enhanced the anticancer effect of doxorubicin. In contrast, tomoroside B increased HIF-1α expression and likely acts as an antioxidant and redox-status modulator. Tomoroside B also synergized with tipifarnib, suggesting that it may enhance the effect of this chemotherapeutic agent by modulating the MAP kinase pathway. In addition, tomoroside A has the potential to inhibit ABCB1 mRNA expression, which is regulated by HIF-1α and is involved in the development of the MDR phenotype in NCI-H460/R cells. Since P-gp efficiently expels doxorubicin from MDR cancer cells, the enhancement of doxorubicin activity in combination with tomoroside A may be due to the inhibition of ABCB1 mRNA expression induced by tomoroside A.

#### 2.3.3. Genus *Glycyrrhiza*

Licorice, scientifically known as *Glycyrrhiza glabra*, belongs to the legume family (Leguminosae or Fabaceae) and is part of a genus comprising over 30 species that are distributed worldwide. It has been historically revered as a highly recommended herb in ancient Egyptian, Roman, Greek, East Chinese, and Western medicinal practices. Licorice root extracts are valued for their many health benefits, e.g., for the treatment of sore throats, tuberculosis, respiratory and liver diseases, as well as for their antibacterial and anti-inflammatory properties. *G. glabra*, which is widely used in Ayurvedic medicine, grows throughout Asia and some parts of Europe. This medicinal plant is particularly common in regions such as Spain, Italy, Turkey, the Caucasus, central Asia, and western China [114]. It is found in Serbia in the Vojvodina province and at the Fruška Gora Mountain [115].

**Scheme 23:**
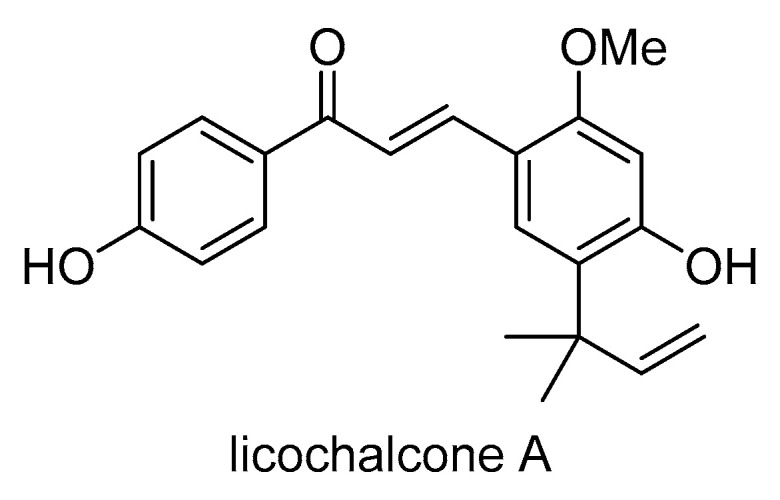
Chemical structure of licochalcone A.

Licochalcone A (Scheme 23), a chalcone extracted from the roots of the Chinese licorice plant *Glycyrrhiza inflata* or *Glycyrrhiza glabra*, is known for its strong anticancer and antioxidant properties. Licochalcone A was found to increase the accumulation of the P-gp substrates daunorubicin and rhodamine 123 in human MDR1 gene-transfected KB/MDR1 cells in a concentration-dependent manner [116]. Licochalcone A also stimulated the ATPase activity of P-gp and inhibited TNF-α-induced NF-κB activation. Additionally, licochalcone A sensitized KB/MDR1 cells to vinblastine, indicating its potential to reverse MDR. These results suggest that licochalcone A may improve the efficacy of cancer chemotherapy through the dual inhibition of P-gp and NF-κB activation.

#### 2.3.4. Genus *Cullen*

*Cullen*, a genus of legumes from the Fabaceae family, is native to tropical, subtropical and arid regions in Africa, Asia and Australia. The genus *Psoralea* is limited to only 20 species, which are mainly found in the Mediterranean regions and southern Africa, while the remaining species belong to other genera. The genus *Cullen* has expanded considerably to include the remaining six species of *Psoralea* found in Africa. A total of 35 *Cullen* species are now known, whose distribution area extends from India and Sri Lanka to the Philippines and Papua New Guinea [117].

*Psoralea corylifolia* or *Cullen corylifolium*, is native to northeastern Africa, the southern Arabian Peninsula, and tropical and subtropical Asia. This plant is important in both Indian and Chinese traditional medicine. Its seeds are rich in various coumarins, including psoralen [118].

**Scheme 24:**
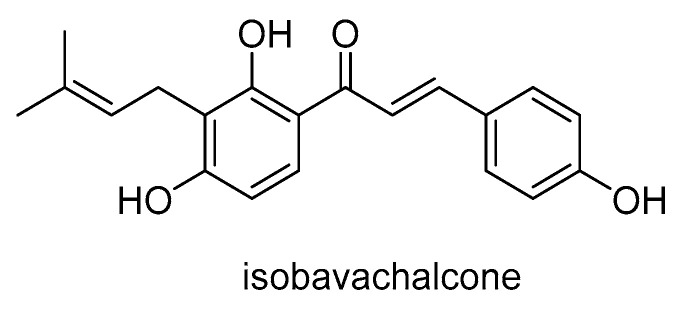
Chemical structure of isobavachalcone.

Isobavachalcone (Scheme 24), a natural flavonoid extracted from the medicinal plant *Psoralea corylifolia*, exhibits significant pharmacological activities on cancer cells via several mechanisms. It induces the production of ROS, inhibits the AKT, ERK and Wnt signaling pathways and promotes apoptosis of cancer cells via mitochondrial or endoplasmic reticulum signaling pathways. Isobavachalcone also has an anti-inflammatory effect by inhibiting the NF-κB signaling pathway and activating the NRF2/HO-1 signaling pathway, thereby reducing the production of various inflammatory mediators [119]. Isobavachalcone has been shown to interact with cell membranes and inhibit the function of the P-gp transporter [120]. It increases the intracellular accumulation of doxorubicin in doxorubicin-resistant human adenocarcinoma colon cancer cells (HT29/Dox), although less effectively than verapamil.

#### 2.3.5. Genus *Artemisia*

The genus *Artemisia* is discussed in Section 2.1.1. *Artemisia absinthium*, also known as common wormwood, is native to North Africa and the temperate regions of Eurasia [121] and is widely naturalized in Canada and the northern United States. It is grown as an ornamental plant and used as an ingredient in the spirit absinthe and some other alcoholic beverages. *A. absinthum* is widespread throughout Serbia and thrives in various habitats such as roadsides, gardens and fields [122].

**Scheme 25:**
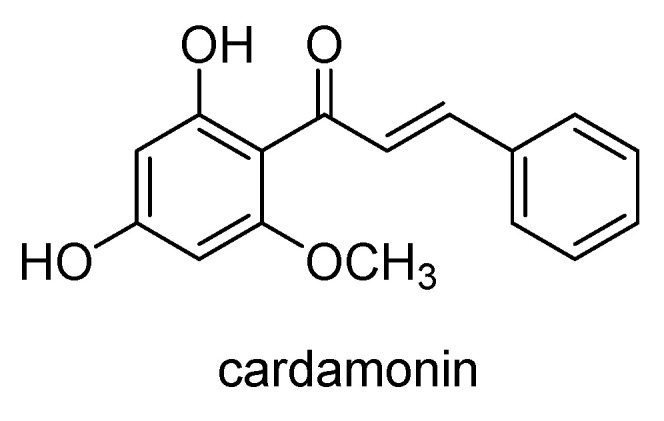
Chemical structure of cardamonin.

Cardamonin (Scheme 25), a natural chalcone firstly isolated from the flowers of *Artemisia absinthium* and found in different plants of the *Zingiberaceae* family, was shown to have an antitumor effect on 5-fluorouracil-resistant gastric cancer cells [123]. The study found that cardamonin significantly increased the chemosensitivity of 5-fluorouracil in cancer cells by suppressing the Wnt/β-catenin signaling pathway. Cardamonin, alone or in combination with 5-fluorouracil, induced apoptosis and cell cycle arrest in resistant cancer cells and decreased the expression of P-gp, β-catenin and TCF4. It specifically disrupted the β-catenin/TCF4 complex and inhibited TCF4-mediated transcription. In a xenograft mouse model, the co-administration of cardamonin and 5-fluorouracil significantly inhibited tumor growth, suggesting that cardamonin is a potential therapeutic strategy for drug-resistant gastric cancer.

#### 2.3.6. Genus *Humulus*

The exact origin of hops is still uncertain, but the presence of three species of the genus *Humulus* (*H. lupulus*, *H. yunnanensis* and *H. japonicus*) in China suggests that hops likely originated in Asia and later spread eastwards to North America and westwards to Europe [124].

*Humulus lupulus* L., known worldwide as an important ingredient in the brewing industry, uses its female inflorescences, hop cones. These cones are rich in polyphenolic compounds and acylphloroglucides, which are crucial for the preservation of beer and for its distinctive smell and taste. In addition, hop cones have long been used in medicine, particularly as a mild sedative to treat insomnia and to stimulate gastric function [124].

**Scheme 26:**
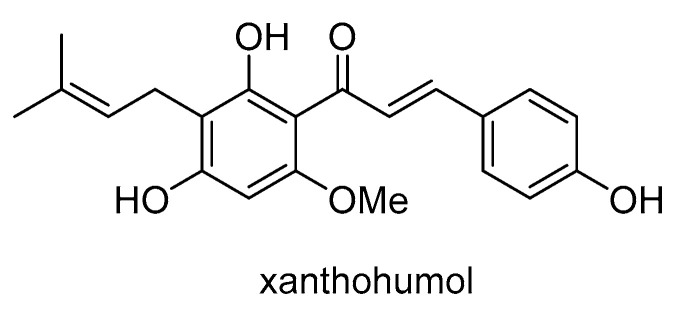
Chemical structure of xanthohumol.

Xanthohumol (Scheme 26) is a natural prenylated chalconoid found in the female inflorescences of *Humulus lupulus* L. Xanthohumol was found to increase the sensitivity of doxorubicin-resistant breast cancer cells (MCF-7/ADR) to doxorubicin by reducing cell viability and stemness [125]. The study found that xanthohumol inhibits the efflux function of P-gp by acting as its substrate and stimulating its ATPase activity. Xanthohumol binds to the central transmembrane domain of P-gp, which overlaps with the doxorubicin binding site and has a stronger binding affinity than doxorubicin. This binding reduces protein- and ligand-position fluctuation, abolishing drug resistance by inhibiting P-gp-mediated doxorubicin transport. Xanthohumol was also found to have the potential to reverse MDR in human KB carcinoma cells by inhibiting P-gp and NF-κB activation [116]. In MDR1 gene-transfected KB/MDR1 cells, it increased the accumulation of P-gp substrates, stimulated P-gp ATPase activity, inhibited TNF-α-stimulated NF-κB activation, and sensitized the cells to the cytotoxicity of vinblastine.

### 2.4. Riccardins

Riccardins are a group of macrocyclic bisbibenzyls extracted from liverworts that have garnered attention for their significant antitumor activity in the literature. They have been found in liverworts such as *Monoclea forsteri*, *Dumortiera hirsuta*, *Plagiochila cristata*, *Lunularia cruciata* and others [126]. They were first discovered in higher vascular plants, *Primula veris* subsp. *macrocalyx,* in 2007 [127]. This was the first case of bisbibenzyls being found in higher plants. The same group confirmed these findings [128], and later, Novaković et al., also discovered bisbibenzyls including riccardins in Serbian *Primula* species: *P. veris* subsp. *columnae* and *P. acaulis* [129].

**Scheme 27:**
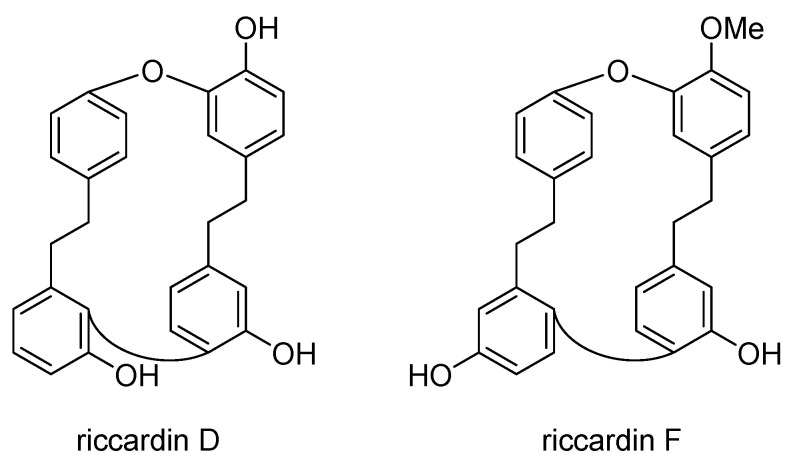
Chemical structures of riccardin D and F.

Studies have demonstrated that riccardins induce apoptosis and inhibit the proliferation of various cancer cell lines, including MDR variants, by modulating the expression and activity of P-gp. Riccardin D (Scheme 27) is a macrocyclic bisbibenzyl initially isolated from the liverwort *Monoclea forsteri* and later found in *Dumortiera hirsuta* and *Plagiochila cristata*. It has exhibited proliferation inhibitory activity and the ability to induce apoptosis in human leukemia cell lines such as HL-60 and the MDR variant K562/A02 [130]. Compared to etoposide, it has demonstrated a stronger inhibition of the DNA relaxation activity of topoisomerase II and reduced P-gp expression in resistant cells. Moreover, riccardin D has been shown to inhibit angiogenesis in lung carcinoma cells, induce apoptosis and autophagy in osteosarcoma cells, suppress the NF-kB signaling pathway, cause DNA damage in PC-3 prostate cancer cells, and induce apoptosis in human leukemia cells through the caspase signaling pathway. These findings suggest its potential as a therapeutic agent against various cancers.

Riccardin F (Scheme 27), a macrocyclic bisbibenzyl isolated from the liverwort *Plagiochasma intermedium*, showed no direct inhibition of cancer cell growth but significantly reduced the IC_50_ of doxorubicin in the doxorubicin-induced MDR leukemia cell line K562/A02. At non-cytotoxic concentrations, riccardin F enhanced the accumulation of doxorubicin and increased the retention of rhodamine123 in K562/A02 cells, indicating its potential to modulate P-gp transport activity and reverse P-gp-mediated MDR [131].

### 2.5. Diarylheptanoids

Diarylheptanoids have attracted significant attention for their potential to inhibit the growth of cancer cells and overcome drug-resistance mechanisms mediated by P-gp. There is a growing body of research highlighting the therapeutic potential of diarylheptanoids as valuable agents in cancer treatment. *Alnus* genus plants are rich in a variety of secondary metabolites such as diarylheptanoids, terpenoids, flavonoids, phenols, steroids, and tannins. Diarylheptanoids are compounds characteristic of the genus *Alnus* but are also found in the genera *Zingiber*, *Curcuma*, *Alpinia* and *Betula*. These compounds are natural phenolics characterized by two aromatic rings linked together by a heptane chain. Within this group, both acyclic and cyclic forms exist, with cyclic diarylheptanoids being less common. These compounds are synthesized from two C6-C3 units linked together, with an additional carbon originating from malonyl CoA [132,133]. The best-known diarylheptanoid is curcumin, which was isolated for the first time in the 19th century from *Curcuma longa*. Curcumin possesses a wide range of biological activities and is one of the most-studied plant secondary metabolites. Diarylheptanoids exhibit antioxidative [134,135], anti-inflammatory [136], antiviral [137], cytotoxic [138,139,140] and anticancer activities [138,139].

#### 2.5.1. Genus *Alnus*

The genus *Alnus*, commonly known as alders, belongs to the family Betulaceae (birch family) and includes over 30 species of trees and shrubs, mainly found in the northern hemisphere and Central America [141]. The largest species in the genus include the red alder (*Alnus rubra*) on the west coast of North America and the black alder (*Alnus glutinosa*), which is native to large parts of Europe and is also cultivated elsewhere. Both species can grow over 30 m high. In contrast, species such as the widespread green alder (*Alnus alnobetula*) and the endemic *Alnus viridis* usually grow as compact shrubs that rarely exceed 5 m in height [142]. *Alnus* species are well-known for their traditional medicinal use, including their application in treating various diseases such as cancer.

*Alnus glutinosa* (L.) Gaertn. (black alder, European alder) is a tree found in Europe, the Mediterranean, southeastern Asia, the Caucasus Mountains, and western Siberia. In the Balkan Peninsula, *A. glutinosa* is found across all Balkan countries—Serbia, Montenegro, Bosnia and Herzegovina, Croatia, and Bulgaria. It typically grows near rivers and creeks and can be found at altitudes up to 1000 m [94]. It often grows with *Fraxinus angustifolia*, *Ulmus laevis*, *Quercus rober*, *Populus alba*, *Salix alba*, *Salix cinerea*, *Rhamus frangula*, *Viburnum opulus* and others. Its Latin name (“glutinosa”) refers to sticky leaves and young branches in spring. *A. incana* (L.) Moench (gray alder) is a deciduous tree (lower than *A. glutinosa*) or shrub found across the northern hemisphere and native to large areas of central and northern Europe. In the Balkan region, it is mainly distributed in Serbia and Albania. The bark of old *A. incana* trees is gray (the reason for the name “gray alder”) and similar to the bark of *Fagus sylvatica*. *A. viridis* subsp. *viridis* is the third *Alnus* species growing in Serbia. It is a 3–5 m tall shrub that grows from 1300 to 2000 m above sea level at the Stara Planina Mountain in Serbia; in Bulgaria; and in Romania in the Carpathian Mountains [94]. It can be also found in the Alps [143].

The bark of *Alnus* species is rich source of diarylheptanoids and triterepnes. *A. glutinosa* and *A. incana* have very similar diarylheptanoid profile, with oregonin, plathyphyloside, rubranoside A and B, hirsutanonol, hirsutanonol-5-β-D-glucopyranoside, alnuside A and B and hirsutenone as the main constituents [144]. Oregonin, (5*S*-1,7-bis(3,4-dihydroxyphenyl)-3-hydroxyheptane-5-O-β-D-xylopyranoside) is an open-chain diarylheptanoid glycoside with 3-carbonyl and 5-xylosyloxy groups and the most-abundant compound in barks of *A. glutinosa* and *A. incana*. This diarylheptanoid has antioxidant, antibacterial, anti-inflammatory and anticancer properties; helps combat obesity; and lowers blood cholesterol and triacylglycerol levels [135,138,145,146,147,148,149,150].

The diarylheptanoid profile of *A. viridis* bark is different in comparison to *A. glutinosa* and *A. incana*. Oregonin, the main diarylheptanoid from black and gray alder, was not found at all in *A. viridis* subsp. *viridis*, and the main diarylheptanoids were platyphylloside and p-hydroxydiarylheptanoids. A high number of diarylheptanoid disaccharides were also found. According to the chemical constituents, *A. viridis* subsp. *viridis* was more similar to *Betula platyphylla* var. *japonica* [151].

A study that compared structurally analogous diarylheptanoids from green and black alder barks collected in Serbia revealed that green alder diarylheptanoids induced significant apoptosis in human non-small cell lung carcinoma cells compared to their black alder analogues [152]. Another study reported the isolation of seven pentacyclic triterpene derivatives (five new) from the bark of green alder from Serbia, and pentacyclic triterpenoids with C-27 hydroxymethyl group were discovered for the first time in genus *Alnus*. These compounds were cytotoxic toward various cancer cells and were selected for an in silico investigation as potential inhibitors of topoisomerases I and IIα [153].

In another study, diarylheptanoids from black alder were found to have chemo-protective effects against doxorubicin in normal keratinocytes [150]. Additionally, various diarylheptanoids from black alder bark, including oregonin, platyphylloside, alnuside A, and others, showed protective effects against DNA damage in human lymphocytes [151].

**Scheme 28:**
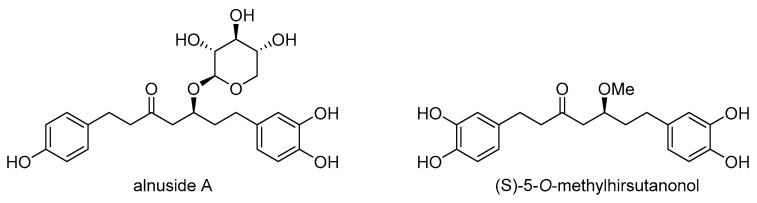
Chemical structures of alnuside A and (S)-5-*O*-methylhirsutanonol.

Furthermore, diarylheptanoids from black alder exhibited protective effects against chemotherapy-induced damage in various cell types, including human non-small cell lung cancer cells and keratinocytes [154]. Noteworthy diarylheptanoids such as platyphylloside, alnuside A, alnuside B, methylhirsutanonol, and hirsutenone, isolated from *Alnus glutinosa*, demonstrated significant anticancer potential, especially against MDR non-small cell lung carcinoma NCI-H460/R cells [155]. While the cytotoxic effects of these diarylheptanoids were not affected by the presence of the MDR phenotype, only alnuside A and (*S*)-5-*O*-methylhirsutanonol (Scheme 28) suppressed P-gp activity. These compounds increased the accumulation of the P-gp substrate doxorubicin in a similar manner to curcumin, indicating that they may modulate P-gp function.

#### 2.5.2. Genus *Curcuma*

**Scheme 29:**
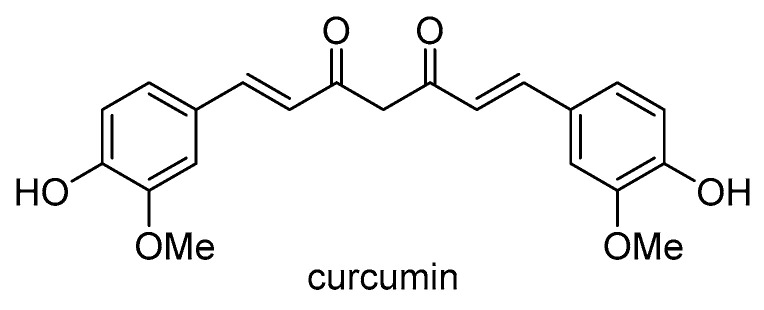
Chemical structure of curcumin.

The genus *Curcuma* is discussed in Section 2.1.2. Curcumin (Scheme 29) is one of the most extensively investigated plant compounds ever. It originates from the rhizomes of *Curcuma longa* and has been extensively studied over the past two decades for its anticancer properties. Traditionally used as a spice, pigment, and in cosmetics, curcumin is known for its anti-inflammatory, antiviral, and anticancer effects, as well as its potential benefits in neurodegenerative, cardiovascular, pulmonary, metabolic, autoimmune, and neoplastic diseases [30,156]. It has also been found to prevent liver damage and lower glucose and cholesterol levels. Curcumin exhibits inhibitory effects on P-gp, MRP1, and BCRP activity, overcomes multidrug resistance (MDR) in cancer cells and tumors, and possesses chemosensitizing properties [157,158,159]. Additionally, curcumin reduces the expression of the ABCB1 gene and sensitizes resistant cancer cells to vincristine by promoting apoptosis through the activation of caspase-3 [160]. Moreover, curcumin is known to modulate the expression of genes involved in DNA repair, inhibit the mTOR signaling pathway, suppress tumor growth, induce apoptosis, and influence the growth and survival of cancer cells [161,162]. Several delivery methods, such as nanoparticles, liposome encapsulation, and curcumin–phospholipid complexes, have been developed to enhance its bioavailability and efficacy in the treatment of resistant cancers [30,163,164].

**Scheme 30:**
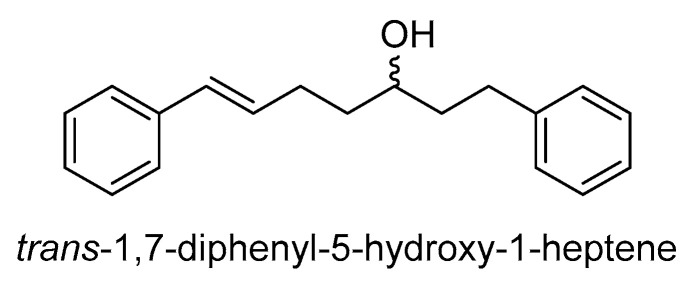
Chemical structure of trans-1,7-diphenyl-5-hydroxy-1-heptene.

A study found that the compound trans-1,7-diphenyl-5-hydroxy-1-heptene (DHH) (Scheme 30), derived from another *Curcuma* species, *C. comosa,* is toxic to drug-resistant human leukemia K562/ADR cells without affecting P-gp expression [165]. The study revealed that when DHH and doxorubicin were used together, the amount of doxorubicin needed to kill the cells decreased significantly, indicating a change in the MDR characteristics. Furthermore, a low dose of DHH increased cell sensitivity to doxorubicin in resistant cells that express P-gp. DHH’s inhibition of P-gp-mediated drug removal led to increased cell death in K562/ADR cells. These findings suggest that DHH could be a new modifier of P-gp function, enhancing drug accumulation and promoting cell death in doxorubicin-resistant leukemia cells.

**Table 1:** Natural products and their derivatives with inhibitory activity against P-gp and MDR cancer cells: Classification and genus origin.

Classes of Natural Products	P-gp Inhibitors and MDR Modulators	Derivatives	Additional Biological Activities *	Origin
sesquiterpenoids	artemisinin [48]	artesunate 4-aryl-2-aminopyrimidines and artesunate- hybrids [53] artesunate-podophyllotoxin conjugate [55]	antimalarial [50] glycolysis inhibition [52] G_2_/M cell cycle arrest and apoptosis [55]	Genus *Artemisia*
	β-caryophyllene [56]	β-caryophyllene oxide [56]	anti-hepatoma effect [56]	
	α-turmerone [58] furanodiene [59] β-elemene [60,61]		anti-angiogenic effect [59] reactive oxygen species (ROS) production [59] DNA strand breaks [59] Apoptosis [59] anti-leukemic effect [60] anti-metastatic effect [61] modulation of signaling pathways [61]	Genus *Curcuma*
	umbelliprenin [64] farnesiferol B [64] farnesiferol C [64] lehmferin [64]			Genus *Ferula*
	costunolide [66,67] ergolide [68] sesquiterpenoid 3 [69]		inhibition of PI3K/AKT signaling pathway [66] anti-leukemic effect [68]	Genus *Inula*
	isopetasin [72] S-isopetasin [72]		increased mitochondrial workload [72] ATP production [72] ROS formation [72] cell death induction [72]	Genus *Petasites*
	dihydro-β-agarofurans [74]			Genus *Celastrus*
diterpenoids	sclareol [78,89]	hybrids of sclareol and doxorubicin [83] hybrids of sclareol and 1,2,4-triazolo[1,5-*a*]pyrimidines [89]	apoptosis [78,89] collateral sensitivity [83] anti-glioblastoma effect [83,89]	Genus *Salvia*
	nicaeenin F [92] nicaeenin G [92] jatrophane diterpenoid 8 [93] jatrophane diterpenoid 9 [93] euphodendrophane A [96] euphodendrophane B [96] jatrophane diterpenoids 10 [97] jatrophane diterpenoids 11 [97] euphoresulan H [98] heliosterpenoids A [100] heliosterpenoids B [100] jatrophane diterpenoid ES2 [101] euphosporophane I [102] jatrophane diterpenoid 12 [103] euphoglomueruphane K [104] euphoglomueruphane L [104]	jatrophane diterpenoid derivative 13 [103] jatrophane diterpenoid derivative 14 [105]		Genus *Euphorbia*
chalcones	flavokawain A [108] flavokawain B [109]		apoptosis [108] inhibition of PI3K/Akt signalling [108]	Genus *Piper*
	tomoroside A [113] tomoroside B [113]		TopoIIα inhibtion [113] HIF1α inhibition [113] MAP kinase inhibition [113]	Genus *Helichrysum*
	licochalcone A [116]		TNF-α inhibition [116] NF-κB activation [116]	Genus *Glycyrrhiza*
	Isobavachalcone [120]		ROS production [119] inhibits the AKT, ERK and Wnt signaling pathways [119] apoptosis [119] anti-inflammatory effect [119] NF-κB signaling pathway inhibition [119] NRF2/HO-1 signaling pathway activation [119]	Genus *Cullen*
	Cardamonin [123]		inhibition of Wnt/β-catenin signaling pathway [123] decreased expression of β-catenin and TCF4 [123]	Genus *Artemisia*
	xanthohumol [116]		reduction of cell viability and stemness [125] inhibition of TNF-α-stimulated NF-κB activation [116]	Genus *Humulus*
riccardins	riccardin D [130] riccardin F [131]		inhibition of NF-κB signaling pathway [130] apoptosis [130] autophagy [130] anti-angiogenic [130] DNA damage [130]	Genera *Lunularia*, *Monoclea*, *Dumortiera*, *Plagiochila*, and *Primula*
diarylheptanoids	alnuside A [144] (S)-5-*O*-methylhirsutanonol [155]		DNA protection [151]	Genus *Alnus*
	Curcumin [160] trans-1,7-diphenyl-5-hydroxy-1-heptene (DHH) [165]		anti-inflammatory effects [30,156] antiviral effects [30,156] anticancer effects [30,156] apoptosis [161,162] mTOR inhibition [161,162]	Genus *Curcuma*

* Only additional biological activities are reported within this review paper.

## 3. Novel Perspectives with Naturally Derived P-Glycoprotein Inhibitors

The development of effective chemotherapeutics with strong and selective anticancer activity is an ongoing need to combat drug resistance. P-gp expression in various cancers has led to extensive research into creating efflux inhibitors capable of overcoming MDR in experimental models. Despite significant research efforts, a clinically effective inhibitor has not yet been discovered. This has shifted focus to alternative strategies, such as bypassing the transporters or exploiting the collateral sensitivity of MDR cells.

Recent discoveries have shown that it is possible to reverse resistance and counteract the selective advantage of resistant cells. Compounds selective to MDR have been found to target ABC-transporter-overexpressing MDR cancer cells by exploiting the vulnerabilities caused by transporter overexpression. Certain natural compounds exhibit preferential toxicity against MDR cells [166].

The development of hybrid compounds comprising a natural-product scaffold and a drug-like fragment (privileged structural motif) with distinct mechanisms of action holds significant value for enhancing the pharmacokinetic and pharmacodynamic properties of potential drug candidates. Recent studies have shown the promising potential of hybrid compounds in cancer treatment, particularly in addressing MDR and enhancing anticancer effects [53,83,89]. The ability of hybrid compounds to overcome MDR by inhibiting P-gp activity and their potential for targeted drug delivery makes them valuable in developing more-effective cancer therapies. The hybrid compounds also showed promising anticancer activities, demonstrating improved selectivity towards MDR cells, indicating their potential to surpass drug resistance. This is a particularly interesting phenomenon referred to as collateral sensitivity. The dual mechanism of action (collateral sensitivity and P-gp inhibition) makes the hybrid compounds potentially valuable for further development in cancer treatment. Overall, the development of hybrid compounds presents a compelling avenue to advance cancer treatment and improve patient outcomes.

One potential way to successfully introduce P-gp inhibitors into clinical practice is to ensure that these inhibitors affect P-gp function in cancer cells without interfering with the P-gp physiological function in important barriers such as the placenta and the blood–brain barrier. Our recent unpublished data have revealed derivatives of natural products that are capable of acting in this way. Therefore, developing natural-inspired drugs with collateral sensitivity, which can evade the P-gp mechanism of resistance and spare normal P-gp function in the body, holds the potential to create highly efficient anticancer agents. These agents will not only possess anticancer properties but also have the ability to enhance traditional chemotherapy effects.

## Data Availability

No new data were created or analyzed in this study.
